# Building local capacity in operational research: a case study in Nepal and India

**Published:** 2023-01-30

**Authors:** Suzanne S Gilbert, GVS Murthy, Kenneth L Bassett, Operational Research Capacity Building Study Group bit.ly/ORCB-study

**Affiliations:** Senior Director, Research & Strategic Opportunities: Seva Foundation, Berkeley, USA.; Director: Indian Institute of Public Health, Hyderabad, India and Professor: London School of Hygiene and Tropical Medicine, London, UK.; Program Director, Seva Canada and Director: British Columbia Centre for Epidemiology and International Ophthalmology, Vancouver, Canada.

## Abstract

A programme of mentor support and training has enabled eye teams in Nepal and India to carry out research to improve their own delivery of eye care services.

Operational research provides eye care personnel with evidence they can use to improve the equity, efficiency, and effectiveness of health services and systems.^1^

Operational research builds on and uses monitoring and evaluation infrastructure, including routine administrative data and quality assurance programmes. It is relevant to almost all aspects of hospital and outreach services: reducing waiting times for cataract surgery, to testing the best ways of counselling patients to improve referral from a screening location. It does not include clinical research.

In 2019, the Indian Institute of Public Health – Hyderabad (IIPHH), together with Seva Foundation and Seva Canada (Seva), both international non-governmental organisations, launched the Operations Research Capacity Building (OCRB) programme. The goal of the programme was to strengthen operational research among four hospitals (three in Nepal and one in India) through a spectrum of activities and research projects, designed to be both opportunistic (to reflect immediate eye programme needs), and strategic (to optimise operational research capacity building, e.g., in health services, health systems, human resources, and public health).

The expected outcomes of the programme were as follows:

1. Eye hospitals would develop capacity to conduct operational research and experience the importance of evidence-informed practice.
2. Hospital management would understand the need for investing in operational research and provide dedicated resources for it.
3. Some of the partner hospitals would become research resource centers for their country or region.

The four hospitals who were invited to join had been involved with Seva previously. They all had an established appetite for research, support from their eye hospital leadership, and had at least one investigator in the team who had research skills at graduate level (e.g., MSc or PhD).

**Figure 1:**
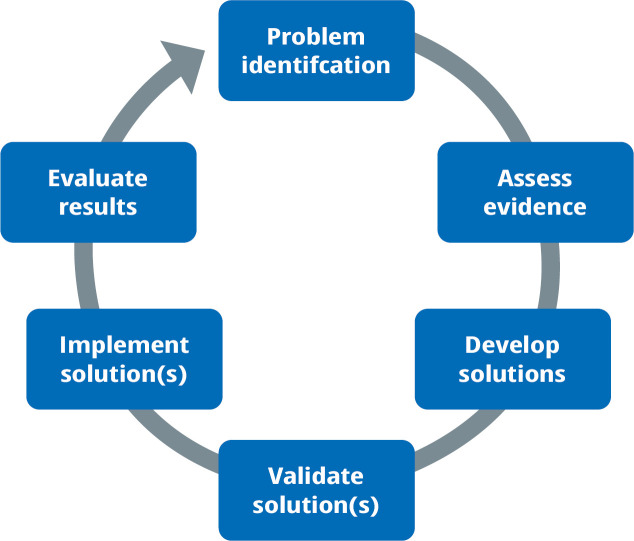
Operational research cycle

## Mentorship model

Each interdisciplinary eye hospital team was assigned a dedicated mentor (from IIPHH) and a support person (from Seva) who consulted with the hospital-based team once a month, via Zoom. These sessions enabled the local teams to develop the skills needed to carry out the steps in the operational research cycle (Figure 1):

- analyse the root causes of any difficulties (through problem tree analysis)
- identify a research question
- conduct literature reviews
- create specific objectives
- finalise the research methodology, including sampling method and sample size estimation
- obtain ethical approval
- design data capture tools and code sheets
- implement the study
- conduct analyses
- prepare manuscripts for publication.

Additional support with data management, statistical analysis, and presentation of data was provided by a dedicated team based at IIPHH.

**Figure 2:**
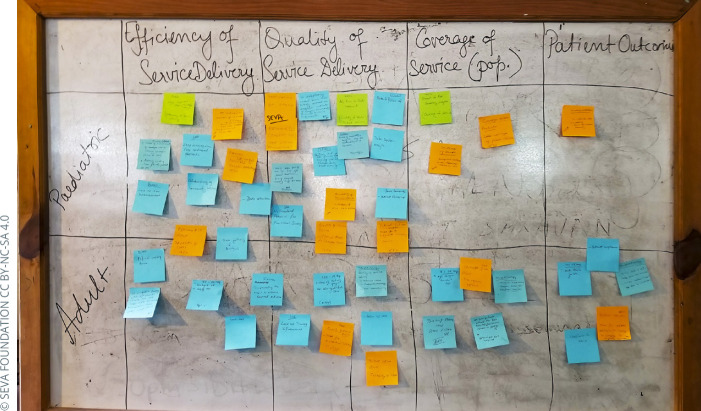
At one of the workshops, the research teams generated ideas for operational research topics by category, e.g., efficiency of service delivery, quality of service delivery, coverage of service (relative to the population), and patient outcomes.

The partner hospital teams also attended a series of structured workshops (Figure 2), provided by a panel of research experts with expertise in a range of different disciplines.

Every two months, each of the four hospital teams gave a virtual progress update to one another and to the panel of research experts. Between these sessions, the dedicated mentors and support persons from IIPHH and Seva, respectively, met to refine next steps.

### Outcomes

The mentorship process was resource intensive but yielded excellent results: to date, three of the four teams have published their research protocols in peer reviewed journals, and the fourth team's manuscript has been submitted and is under review.

**Figure 3:**
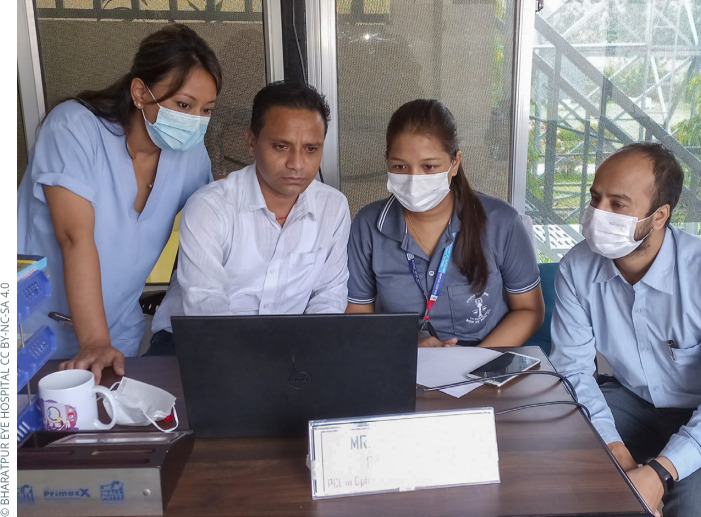
Gopal Bhandari shares STATA workshop learnings with Bharatpur Eye Hospital team members

The participating institutions have become proficient in the key steps needed to carry out operational research, such as statistical analysis. Several of the hospital teams have started workshops and processes within their institutions designed to improve the quality of data collected and its systematic use in programme management, including sharing data analysis skills learnt during the workshops with team members at the hospital (Figure 3).

The operational challenges addressed by the four hospital teams are given below, alongside feedback from their team leaders.

**Bharatpur Eye Hospital, Chitwan, Nepal:** Improving the follow-up rates for pediatric department patients who are advised to return for follow-up visits.^2^

“This research training really helped me on the personal and professional level. Now, when I think about any problem, I think about the solution for the same. Initially I used to think research needs to be some big topic. But the day-to-day activities that we are doing, thinking about a new way to do it, also is research. We have set up a research team and have started training our internal staff as well as trying to build programs for other eye hospitals.”

– Manisha Shrestha, Pediatric Ophthalmologist

**Reiyukai Eiko Masunaga Eye Hospital, Banepa, Nepal:** Increasing the volume and uptake of retinal services (screening and treatment) through patient referrals from general community hospitals.^3^

“Through this workshop I learned how to identify a problem and analyse it. The research training and coaching was a great help to us. Through our intervention with the local general hospital, the number of referred people with Diabetic Retinopathy increased and there was a quite significant change in the knowledge of healthcare professionals. The project has brought a lot of changes in how our own team works.”

– Ruchi Shrestha, Medical Director and Vitreo-retinal Surgeon

**Dr Shroff's Charity Eye Hospital, New Delhi, India:** Determining the effect of screening and generating awareness in its three-million-person service area through a door-to-door intervention to increase the use of community-based vision centres.^4^

“During our research project the team learned about the seriousness of keeping good data and collecting different data points which may be contributing to the results in an indirect way. I think the way to go ahead is to make research like a culture. Have a group of people who are interested in starting small projects. We can make it a habit to collect that baseline data, which is important for comparison, and then see the impact in a very scientific way.”

– Shalinder Sabherwal, Head– Department of Community Ophthalmology and Public Health Research

**Lumbini Eye Institute and Research Centre, Siddharthanagar, Nepal:** Improving timely diabetic patient referral flow and compliance from peripheral eye centres to the main hospital.

“I have had a few publications and I was happy with it. This workshop gave me a deeper understanding of what research actually is. It has lead me to want to experience more. At the institutional level this project strengthened tracking of patient referrals which is very important for a tertiary setting. I feel very fortunate to be part of this team.”

– Binita Bhattarai, Associate Professor and Oculoplastic Surgeon

## Future plans

The ORCB programme underscored the value of a long-term partnership and mentorship model in developing research skills in eye hospitals. While labour intensive, this level of long-term commitment seems necessary for programme success.

Two online courses are being developed as part of Seva's e-Learning platform, InSight, to make evidence competency a part of the skill development library. These courses will be accessible to eye care workers (anytime, anywhere) with less intensive mentoring input; they include a module on evidence-informed practice and an intermediate-level blended learning path for practitioners. For more information, please email Insight@seva.org

*The authors would like to acknowledge the valuable contributions of all members of the Operational Research Capacity Building Study Group:* bit.ly/ORCB-study
